# A start codon mutation of the FRMD7 gene in two Korean families with idiopathic infantile nystagmus

**DOI:** 10.1038/srep13003

**Published:** 2015-08-13

**Authors:** Jae-Hwan Choi, Jin-Hong Shin, Je Hyun Seo, Jae-Ho Jung, Kwang-Dong Choi

**Affiliations:** 1 Department of Neurology, Pusan National University School of Medicine, Research Institute for Convergence of Biomedical Science and Technology, Pusan National University Yangsan Hospital, Yangsan, Korea; 2 Department of Ophthalmology, Pusan National University School of Medicine, Research Institute for Convergence of Biomedical Science and Technology, Pusan National University Yangsan Hospital, Yangsan, Korea; 3 Department of Neurology, Pusan National University Hospital, Pusan National University School of Medicine and Biomedical Research Institute, Busan, Korea

## Abstract

Idiopathic infantile nystagmus (IIN) is the involuntary oscillation of the eyes with onset in the first few months of life. The most common form of inheritance is X-linked, and mutations in FRMD7 gene are a major cause. To identify the FRMD7 gene mutations associated with X-linked IIN, we performed PCR-based DNA direct sequencing in 4 affected subjects from 2 Korean families. We also assessed structural abnormalities of retina and optic nerve head using optical coherence tomography (OCT). Genetic analysis revealed a A>G transversion at nucleotide c.1, the first base of the start codon. This mutation leads to the loss of the primary start codon ATG for methionine, which is replaced by a triplet GTG for valine. The alternative in-frame start codon is not present around a mutation. OCT revealed the morphological changes within the optic nerve head, including shallow cup depth and small cup-to-disc ratio. In summary, we identified a novel start codon mutation within the FRMD7 gene of 2 Korean families. Our data expands the mutation spectrum of FRMD7 causing IIN. We also demonstrated abnormal developments of afferent system in patients with FRMD7 mutations using OCT, which may help to understand the etiological factor in development of nystagmus.

Idiopathic infantile nystagmus (IIN) is the involuntary oscillation of the eyes with onset in the first few months of life^1^. IIN usually develops independently of any other ocular diseases such as albinism, achromatopsia or Leber congenital amaurosis. Many hypotheses have been proposed to explain the mechanism of IIN, including miswiring of retinal ganglion cells^2^, the instability of the neural integrator^3^ or disrupted development of a dual pursuit/motion tracking systems^4^. However, each hypothesis alone is insufficient to explain the variable phenotype of IIN.

IIN may be inherited as an autosomal dominant, autosomal recessive or X-linked trait. The most common form of inheritance is X-linked, which can be either dominant or recessive. The only known gene is the FRMD7 located at Xq26.2^5^,^6^. The other X-linked loci have been identified at Xp11.4-p11.4-p11.3 and Xp22, but the responsible gene at the p11.4-p11.3 locus has not yet been identified. GPR143 (G protein-coupled receptor 143) at Xp22 is primarily linked to ocular albinism type 1. Many mutations within FRMD7 gene have been reported in IIN patients, and discovery of gene mutations has led to understand the etiology of IIN at a cellular level. Previous expression studies suggested that FRMD7 may play an important role during neuronal development, specifically elongation of axons and dendrites^5^,^7^,^8^. *In situ* hybridization studies showed restricted FRMD7 expressions in the developing vestibulo-ocular reflex and optokinetic reflex arcs, including the neural retina, optic stalk, vestibular apparatus, and cerebellum^5^,^9^. Recently, optical coherence tomography (OCT) study revealed abnormal developments of retina and optic nerve in patients with FRMD7 mutations^10^.

In this study, we report a common start codon mutation of FRMD7 gene in 2 Korean families. We also assessed structural abnormalities of retina and optic nerve head using OCT.

## Results

### Clinical characteristics

**Figure 1:**
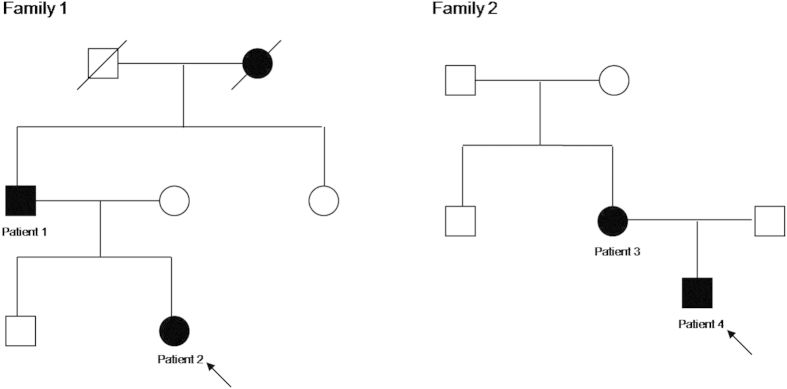
Pedigree of the family. Solid symbols (squares = males, circles = females) indicate clinically affected individuals; open symbols, unaffected individuals; and slashed symbols, deceased individuals. The affection status of the deceased individuals was obtained historically from other members.

Clinical features, ophthalmic and oculographic findings of the patients are described in Table 1 (Fig. 1). The onset of nystagmus was within the first 6 months in all. Two patients (patient 1 and 2) reported dizziness or oscillopsia. Two (patient 1 and 3) described head oscillation in the horizontal plane during the attempt to concentrate on a task or read a book. Three (patient 1, 2, and 3) showed an abnormal head posture, and none had strabismus. The visual acuities ranged from 0.4 to 1.0 and color vision was normal.

All patients showed mainly horizontal, conjugate nystagmus in primary position. The nystagmus waveform was pendular in 2 (patient 1 and 4) and jerky in the other 2 (patient 2 and 3). Without visual fixation, the nystagmus decreased in 2 patients (patient 3 and 4) and changed its direction in 1 patient (patient 2). All patients showed gaze-evoked nystagmus (GEN) and reversed optokinetic nystagmus. In 2 patients (patient 1 and 4), the nystagmus decreased on convergence. The patterns and waveforms of nystagmus were different even among patients from the same family.

### Mutation detection in FRMD7

**Figure 2:**
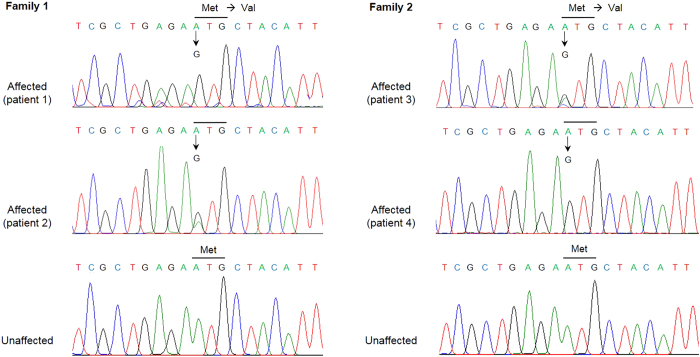
Sequencing results of affected and unaffected individuals. The chromatograms show a start codon mutation (c.A>G, p.M1>V) in 2 hemizygous males (patient 1 and 4) and heterozygous females (patient 2 and 3) of each family.

Sequence analysis revealed a A>G transversion at nucleotide c.1, the first base of the start codon in 2 hemizygous males (patient 1 and 4) and heterozygous females (patient 2 and 3) (Fig. 2). This mutation leads to the loss of the primary start codon ATG for methionine, which is replaced by a triplet GTG for valine. Our start codon mutation is not listed either in public variant database nor in scientific literature, and is predicted to cause truncating mutation. No sequence change was observed in the remaining coding sequence of FRMD7. No mutation was detected in any of unaffected members or in the 100 normal controls.

### OCT findings

**Figure 3:**
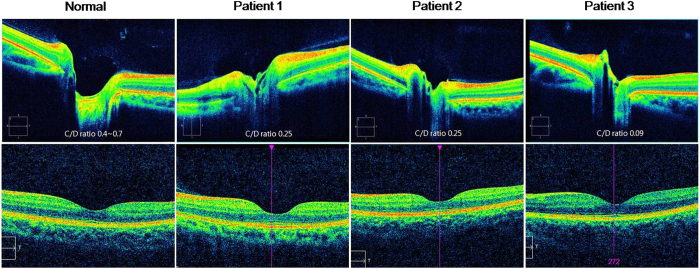
Optical coherence tomography scans of patients. The optic nerve cup is shallow with decreased cup volume in 3 patients (upper pannel). The cup-to-disc ratio is below the normal range (0.4 ~ 0.7) in each patient. The fovea was grossly normal (lower panel).

In optical coherence tomography, the optic nerve cup was shallow with decreased cup volume in all patients (Fig. 3, upper panel). In each patient, the CDR was below the normal range (0.4 ~ 0.7). The retinal nerve fiber layer thickness was within the normal range in all. The fovea was grossly normal without evidence of foveal hypoplasia including the extrusion of inner retinal layer, shallow foveal pit, ONL widening, and OS lengthening (Fig. 3, lower panel).

## Discussion

The FRMD7 gene maps to chromosome Xq26.2 and comprises 12 exons. The 714-amino acid FRMD7 protein contains an N-terminal FERM domain and FERM-adjacent (FA) domain. FERM domains have 3 lobed cloverleaf structures, and each lobe (F1, F2, F3) represents a compactly folded structure^6^. FRMD7 expression studies using *in situ* hybridization showed restricted expressions within the developing afferent and efferent arms of vestibule-ocular and optokinetic reflex, the developing cerebellum and neural integrator site^5^,^9^. The functional role of FRMD7 protein is as yet unknown because there have been no detailed functional studies for FRMD7 mutations. However, it shares close homology with FARP1 (FERM, RhoGEF and pleckstrin domain protein 1) and FARP2 that has a role in neuronal development^5^. FRMD7 also promotes neurite elongation at the actin-rich growth cone ends through the modulation of actin cytoskeleton, and knockdown of FRMD7 leads to a significant reduction in overall neurite length^7^,^11^,^12^. A recent study also found an interaction between FRMD7 protein and calcium/calmodulin-dependent serine protein kinase (CASK) that links the plasma membrane to the actin cytoskeleton^13^. Thus, FRMD7 mutations may cause nystagmus by defective axogenesis, dendritogenesis, and neuronal guidance in the areas of the brain that control eye movements. Further functional studies are clearly needed.

In the present study, we identified a common novel mutation, c.1A>G within the FRMD7 gene in 2 Korean families with IIN. This mutation causes the loss of the primary start codon ATG for methionine, which is replaced by a triplet GTG for valine (p.Met1Val). The alternative in-frame start codon is not present around a mutation, and our start codon mutation is expected to cause truncating mutation by frameshift at a downstream sequence. Despite the common start codon mutation in the 2 families, the clinical features and oculographic findings were diverse.

To date, 66 different mutations within FRMD7 have been reported (including the present one. https://sites.google.com/site/molneurol/lsdb/frmd7)^5^,^9^,^10^,^14^,^15^,^16^,^17^,^18^,^19^,^20^,^21^. About half (47%) of the mutations are missense which can render the resulting protein nonfunctional. The other half (53%) are predicted to cause gross defects of FRMD7 protein due to nonsense mutations, frameshift caused by insertion or deletion, and aberrant splicing. Many mutations cluster around the F3 lobe of the FERM domain and the FA domain that are located at residues 179–299 (exon 7–9) and 294–336 (exon 9–11), respectively. This suggests that these regions play important roles in the function of FRMD7. Among the 12 exons of FRMD7, exon 9 represents the most common mutation-rich exon (24%), followed by exon 8 (14%) and 12 (12%). Some mutations have been reported in ethnically different families. The most common reported mutation is c.1003C>T (p.R335X), which has been detected in 4 different families^5^,^19^. Two mutations have been reported in exon 1 of FRMD7, similar to our families. One was a missense mutation (c.47T>C) resulting in the substitution of phenylalanine by serine at position 16^9^,^20^, and the other was an in-frame 3-bp deletion (c.41delAGA) causing the deletion of lysine at position 14^5^,^21^. Both mutations were detected in 2 ethnically different families.

Recently, it was reported that FRMD7 mutations may be associated with abnormal developments of afferent visual systems^10^. FRMD7 mRNA expression was seen in the developing human neural retina and optic nerve, and OCT identified foveal hypoplasia and optic nerve head changes in IIN patients with FRMD7 mutations. Our patients also showed morphological changes within the optic nerve head, such as shallow cup depth and small cup-to-disc ratio. However, the foveal hypoplasia was not prominent in comparison with inherited developmental retinal disorders such as albinism, PAX6 mutations, and achromatopsia. The foveal hypoplasia in previous study was also much milder than albinism^10^. Therefore, these changes may be subclinical, resulting in better visual acuity in patients with FRMD7 mutations, as compared to other retinal defects. Further studies are needed to determine how FRMD7 mutations lead to the abnormal developments of afferent systems, and subsequent effects on the neural circuit within the oculomotor system generating nystagmus.

In summary, we identified a novel start codon mutation, c.1A>G within the FRMD7 gene of 2 Korean families. Our data expands the mutation spectrum of FRMD7 causing IIN. We also demonstrated abnormal developments of afferent system in patients with FRMD7 mutations using OCT, which may help to understand an etiological factor in development of nystagmus.

## Methods

### Patients

Two Korean families with idiopathic infantile nystagmus (IIN) were recruited at the Neuro-ophthalmology Clinic of Pusan National University Yangsan Hospital. Two affected subjects from each family were evaluated (Fig. 1). Patients underwent detailed ophthalmic examinations, eye movement recordings and direct sequence analysis. All experiments followed the tenets of the Declaration of Helsinki, and informed consent was obtained after the nature and possible consequences of this study had been explained to the participants. This study was approved by the Institutional Review Boards of Pusan National University Yangsan Hospital.

### Ophthalmic examinations

Detailed ophthalmic examinations were performed on each subject. The visual acuity was recording using Snellen visual acuity charts. The color vision was evaluated through Ishihara testing. Slit lamp bio-microscopy was performed in the dark to exclude iris transillumination. The presence of strabismus and anomalous head posture were also analyzed.

### Oculographic study

Spontaneous and gaze-evoked nystagmus (GEN) in the horizontal plane were recorded binocularly using 3-dimensional video-oculography (SLMED, Seoul, Korea). The effects of visual fixation, convergence, and optokinetic stimuli were also evaluated.

### Mutation analysis

Mutations in FRMD7 gene were screened by direct sequence analysis using genomic DNA from the patients’ peripheral blood. All coding exons and intron-exon junctions from FRMD7 gene were amplified through polymerase chain reaction (PCR) with the primers listed in Table 2. PCR-amplified products were separated and purified using 2% agarose gel and DNA extraction kit (SolGent, Daejeon, Korea), cycle-sequenced with PCR primers using BigDye Termiator Sequencing Kit and ABI PRISM 3730XL DNA analyzer (Applied Biosystems, Foster, CA, USA). Sequences were manually examined for variants Chromas software. The reference cDNA sequence was obtained from the Genbank database (NM_194277.1), when c.1 corresponds to the first nucleotide of the translation initiation codon. The variant was tested for novelty through searches of literature and public variant database including dbSNP and the comparison with in-house genomic data.

### Optical coherence tomography

Ultrahigh-resolution spectral-domain OCT (Cirrus HD-OCT, Carl Zeiss Meditec, Inc, Dublin, California, USA) was used to acquire tomograms from both eyes in 3 patients (patient 1, 2, and 3). The OCT acquires images at a speed of 27000 A-scans per second and 5 μm axial resolution in tissue. The Macular cube 200 × 200 protocol was used to obtain macular thickness measurement. This scan generates a cube of data by performing raster scanning in a 6 × 6 mm square grid. For optic nerve head acquisition, the fixation spot was altered and the scan window was centered at the optic nerve head using Optic Disc Cube 200 × 200 scan. The acquired images were analyzed using the Cirrus HD-OCT software (version 5.0), and custom scripts in Image J software (Rasband, W.S., ImageJ, U.S. National Institutes of Health, Bethesda, MD, USA).

The foveal and optic nerve head B-scans were segmented and analyzed for morphological abnormalities. Foveal hypoplasia was evaluated by the extrusion of inner retinal layer, widening of the outer nuclear layer (ONL), and lengthening of the cone outer segment (OS)^22^. Optic nerve changes were assessed by cup-to-disc ratio (CDR) and cup depth.

## Additional Information

**How to cite this article**: Choi, J.-H. *et al.* A start codon mutation of the FRMD7 gene in two Korean families with idiopathic infantile nystagmus. *Sci. Rep.* **5**, 13003; doi: 10.1038/srep13003 (2015).

## Figures and Tables

**Table 1:** Clinical characteristics and oculographic findings of the patients.

Patient no.	Age/Sex	Symptoms	Head nodding	Head tilt	Strabismus	Visual acuity(right/left)	Nystagmus		Gaze	Convergence	OKN
							With fixation	Without fixation			
1	38/M	Dizziness	Yes	Right	No	0.6/0.6	Horizontal pendular	No change	GEN (+)	Suppression	Reversed
2	8/F	Oscillopsia	No	Left	No	0.9/0.8	Left-beating horizontal	Direction change	GEN (+)	No change	Reversed
3	35/F	(−)	Yes	Left	No	1.0/1.0	Left-beating horizontal	Suppression	GEN (+)	No change	Reversed
4	4/M	(−)	No	No	No	NP	Horizontal pendular	Suppression	GEN (+)	Suppression	Reversed

M = male; F = female; GEN = gaze-evoked nystagmus; OKN = optokinetic nystagmus.

**Table 2:** Primers and PCR conditions for FRMD7.

Exon	Primer sequence (forward/reverse)	Tm (°C)	Product size (bp)
1	5′-CAGGAGACTGCCCAGATGCTAT-3′	62.9	358
	5′-TTGCTCTCTAATGGGCTGTTCA-3′	62.1	
2	5′-CGTGCTGCAGTATCAGGTTAGG-3′	62.1	243
	5′-TGAACCCTACATACCTAGCTGCAA-3′	62.1	
3	5′-GCCACCTATTTGACATTGCTGT-3′	61.3	278
	5′-TGAGAAATTGTGTGAGGTTGTTGA-3′	61.8	
4	5′-CACAGAAGTCTGTAGGAGGGAGTG-3′	61.6	296
	5′-TGCCCCCAATAAATGGAGAATA-3′	62.5	
5	5′-CTTGGATCTGGGAGAAGGAAAA-3′	61.8	237
	5′-CCTGTGCTTGGTTCTCTACCTG-3′	61.2	
6-7	5′-TGCTCCATTGCTAAGTTCCTCA-3′	62.1	583
	5′-ACACCCAAGTTTGAGCCAAGAT-3′	62.1	
8	5′-TGCACTGTCTTACAAGCCAACTC-3′	61.7	231
	5′-CGATTTGCAGAAACAACCAAAA-3′	62.2	
9	5′-GCATTGGGATTTGAAGGTCTTT-3′	61.5	300
	5′-TCCTAAGCCTCCTGTGTTATTGAA-3′	61.3	
10-11	5′-TTCAACAATAAACGAGGCTTTCA-3′	61.0	480
	5′-CCCCAGGAAGCTAACCTACTCA-3′	61.8	
12a	5′-ACCAAATGGCCTTTTCCTTCT-3′	61.2	685
	5′-ATGTTGCTCCTACCGCTAGTCC-3′	61.9	
12b	5′-ATGTAGAGCCCACTGCAATGAA-3′	61.9	634
	5′-TGCCTAAGTCGGTAACATGGAA-3′	61.7	

## References

[b1] Leigh R. J. & Zee D. S. The neurology of eye movements 4^th^ edn (ed. Gilman S. *et al.* .) Ch. 10, 512–516 (Oxford University Press, 2006).

[b2] Huber-Reggi S. P. *et al.* Severity of infantile nystagmus syndrome-like ocular motor phenotype is linked to the extent of the underlying optic nerve projection defect in zebrafish belladonna mutant. J Neurosci. 32, 18079–18086 (2012). 23238723 10.1523/JNEUROSCI.4378-12.2012 PMC6621732

[b3] Schneider R. M. *et al.* Neurological basis for eye movements of the blind. PloS One. 8, e56556 (2013). 23441203 10.1371/journal.pone.0056556 PMC3575504

[b4] Brodsky M. C. & Dell’Osso L. F. A unifying neurologic mechanism for infantile nystagmus. JAMA Ophthalmol. 132, 761–768 (2014). 24525626 10.1001/jamaophthalmol.2013.5833

[b5] Tarpey P. *et al.* Mutations in FRMD7, a newly identified member of the FERM family, cause X-linked idiopathic congenital nystagmus. Nat Genet. 38, 1242–1244 (2006). 17013395 10.1038/ng1893 PMC2592600

[b6] Watkins R. J., Thomas M. G., Talbot C. J., Gottlob I. & Shackleton S. The Role of FRMD7 in Idiopathic Infantile Nystagmus. J Ophthalmol. 2012, 460956 (2012). 21904664 10.1155/2012/460956 PMC3163398

[b7] Betts-Henderson J. *et al.* The nystagmus-associated FRMD7 gene regulates neuronal outgrowth and development. Hum Mol Genet. 19, 342–351 (2010). 19892780 10.1093/hmg/ddp500

[b8] Self J. *et al.* Frmd7 expression in developing mouse brain. Eye. 24, 165–169 (2010). 19265863 10.1038/eye.2009.44

[b9] Thomas M. G. *et al.* The clinical and molecular genetic features of idiopathic infantile periodic alternating nystagmus. Brain. 134(Pt 3), 892–902 (2011). 21303855 10.1093/brain/awq373 PMC4125620

[b10] Thomas M. G. *et al.* Abnormal retinal development associated with FRMD7 mutations. Hum Mol Genet. 23, 4086–4093 (2014). 24688117 10.1093/hmg/ddu122 PMC4082370

[b11] Diakowski W., Grzybek M. & Sikorski A. F. Protein 4.1, a component of the erythrocyte membrane skeleton and its related homologue proteins forming the protein 4.1/FERM superfamily. Folia Histochem Cytobiol. 44, 231–248 (2006). 17219717

[b12] Pu J. *et al.* FERM domain containing protein 7 (FRMD7) upregulates the expression of neuronal cytoskeletal proteins and promotes neurite outgrowth in Neuro-2a cells. Mol Vis. 18, 1428–1435 (2012). 22690121 PMC3370689

[b13] Wakkins R. J. *et al.* A novel interaction between FRMD7 and CASK: evidence for a causal role in idiopathic infantile nystagmus. Hum Mol Genet 22, 2105–2018 (2013). 23406872 10.1093/hmg/ddt060 PMC3633374

[b14] AlMoallem B. *et al.* Novel FRMD7 Mutations and Genomic Rearrangement Expand the Molecular Pathogenesis of X-Linked Idiopathic Infantile Nystagmus. Invest Ophthalmol Vis Sci. 56, 1701–1710 (2015). 25678693 10.1167/iovs.14-15938

[b15] Zhang X. *et al.* Identification of three novel mutations in the FRMD7 gene for X-linked idiopathic congenital nystagmus. Sci Rep. 4, 3745 (2014). 24434814 10.1038/srep03745 PMC3894538

[b16] Zhu Y. *et al.* Identifcation of a novel mutation p.I240T in the FRMD7 gene in a family with congenital nystagmus. Sci Rep. 3, 3084 (2013). 24169426 10.1038/srep03084 PMC3812648

[b17] Liu Z. *et al.* A novel missense mutation in the FERM domain containing 7 (FRMD7) gene causing X-linked idiopathic congenital nystagmus in a Chinese family. Mol Vis. 19, 1834–1840 (2013). 23946638 PMC3742126

[b18] Radhakrishna U. *et al.* Novel homozygous, heterozygous and hemizygous FRMD7 gene mutations segregated in the same consanguineous family with congenital X-linked nystagmus. *Eur J Hum Genet*. 20, 1032–1036 (2012). 22490987 10.1038/ejhg.2012.60 PMC3449080

[b19] Guo Y. *et al.* Heterogeneous phenotype in a family with the FERM domain-containing 7 gene R335X mutation. Can J Ophthalmol. 49, 50–53 (2014). 24513357 10.1016/j.jcjo.2013.09.001

[b20] Gudzenko S. *et al.* Novel mutations in FRMD7 in Russian families with X-linked congenital motor nystagmus. Genomic Med. 2, 243 (2008).

[b21] Zhang Q., Xiao X., Li S. & Guo X. FRMD7 mutations in Chinese families with X-linked congenital motor nystagmus. Mol Vis. 13, 1375–1378 (2007). 17768376

[b22] Thomas M. G. & Gottlob I. Optical coherence tomography studies provides new insights into diagnosis and prognosis of infantile nystagmus: a review. Strabismus. 20, 175–180 (2012). 23211143 10.3109/09273972.2012.735336

